# Evaluation of FR104, a Treg sparing antagonist anti-CD28 monovalent Fab’ antibody in kidney transplantation in non-human primates

**DOI:** 10.1186/1479-5876-10-S3-P61

**Published:** 2012-11-28

**Authors:** Nicolas Poirier, Nahzli Dilek, Caroline Mary, Jeremy Hervouet, David Minault, Julien Branchereau, Xavier Tillou, Stephanie Le Bas-Bernardet, Bernard Vanhove, Gilles Blancho

**Affiliations:** 1 Institute of Transplantation Urology Nephrology, University of Nantes, INSERM UMR 1064, Nantes, France; 2 Effimune, Nantes, France CHU Nantes, Nantes, France

## Background

Targeting CD28 costimulation with antagonist anti-CD28 antibodies has the potential to block effector T cells without perturbation of the CTLA-4 and PDL-1-mediated inhibitory signals important for the function of Treg cells, which might favour tolerance induction.

## Methods and results

Here we evaluated in a non-human primates this “Treg sparing strategy” with FR104, a novel monovalent humanized and pegylated Fab’ anti-CD28 antibody fragment. PK/PD studies in monkeys revealed that FR104 presented an elimination half-life of 8 days and 100% target saturation over at least a month after a single iv injection of 5 mg/kg. FR104 was next evaluated in a baboon kidney allograft model at the dose of 5 mg/kg at day 0, 4, 14 and then every two-week until 3 months. Monotherapy modestly but significantly prolonged allograft survival (MST: 18.5 days for monotherapy vs 6 days for untreated recipients). FR104 synergized with low doses tacrolimus (lowTac, trough: 5-10 ng/ml; MST >100 days for FR104/lowTac vs. 15 days for lowTac alone) as well as with calcineurin-free regimens: therapeutic doses of MMF or rapamycin (day 0-90) with 1 mg/kg of corticosteroids from day 0-14 (MST >100 days for FR104 + MMF/Rapa vs. 18/15 days for MMF/Rapa alone). Flow cytometry analyses indicated that blood Treg cells of the natural and inducible types were preserved in FR104/MMF or FR104/lowTAC bitherapies and accumulated in FR104 monotherapy and in FR104/Rapa bitherapy, whereas Treg cells were lowered by MMF and lowTac monotherapies. Histology also revealed that CTLA4+ and Foxp3+ T lymphocytes were accumulated into the graft of FR104 treated recipients.

## Conclusion

FR104 presented Treg sparing properties in kidney transplantation and this was associated with prevention of graft rejection in synergy with tacrolimus, MMF or rapamycin.

